# Electrochemical and DFT studies of brass corrosion inhibition in 3% NaCl in the presence of environmentally friendly compounds

**DOI:** 10.1038/s41598-019-52635-2

**Published:** 2019-11-06

**Authors:** Milan B. Radovanović, Žaklina Z. Tasić, Marija B. Petrović Mihajlović, Ana T. Simonović, Milan M. Antonijević

**Affiliations:** University of Belgrade, Technical Faculty in Bor, VJ 12, P.O. Box 50, 19210 Bor, Serbia

**Keywords:** Corrosion, Materials science

## Abstract

The effects of adenine, salicylaldoxime and 4(5)-methylimidazole on brass corrosion in NaCl were investigated. The investigation comprised electrochemical techniques, scanning electron microscopy and quantum chemical calculation. The results obtained by polarization measurements show that the examined compounds successfully inhibited the corrosion of brass. Additionally, the quantum mechanical calculations indicate that there is a correlation between energy gap and inhibition efficiency. Moreover, the inhibition mechanism includes the adsorption of the inhibitor on active sites on the electrode surface, which was confirmed by SEM-EDS analysis of the brass.

## Introduction

Copper and its alloys are used extensively in various industries^1–3^. Brass is widely used in shipbuilding and marine engineering^3,4^ as well as in the manufacture of heat exchangers, due to its corrosion resistance. In spite of the corrosion resistance due to oxide films formed on its surface, brass is prone to dissolution in solutions containing high concentrations of oxygen and chloride, sulfate and nitrate ions^5^. Generally, corrosion can be reduced by controlling the pH or using inhibitors^6^. It is considered that the heterocyclic organic compounds act by forming an insoluble polymeric complex between metal and inhibitor, thereby forming a protective film on the metal surface^3^. Different classes of organic compounds have been investigated as potential corrosion inhibitors of brass in chloride media^7–10^. A characteristic of these compounds is the presence of functional groups containing N, S, and O atoms^11,12^, which give the molecule the ability to overlay a large area of a brass surface. Research has been carried out in the past two decades regarding “green” inhibitors, which have been proven to be cheap and effective molecules with low impact or no impact on the environment^13^.

The aim of this paper is to examine the influence of different concentrations of adenine, salicylaldoxime and 4(5)-methylimidazole on the corrosion of brass in naturally aerated 3% NaCl solution. Adenine is an inexpensive compound that is biodegradable and nontoxic to the environment. Additionally, adenine behaves as a good copper corrosion inhibitor^6,13^. According to the literature, salicylaldoxime is also a good corrosion inhibitor of copper^14^. Due to its application in the food industry^15^, imidazole has attracted the attention of scientists as a corrosion inhibitor in different solutions (HNO_3_, H_2_SO_4_, HCl, NaCl and NaHCl)^16^.

## Materials and Methods

### Electrochemical measurements

The brass (Cu37Zn) working electrode was prepared in the following way: first, a brass wire was cut and then sealed with a material based on methyl methacrylate. The exposed surface area of the electrode was 0.49 cm^2^. The brass electrode was polished with alumina paste (0.3 μm Al_2_O_3_, Buehler USA), washed with distilled water and dried prior to each measurement.

A potentiostat (IVIUM XRE, IVIUM Technologies) with suitable software was applied for electrochemical tests. The system consisted of three electrodes, brass, platinum and standard calomel electrode (SCE), as the working, auxiliary and reference electrodes, respectively.

Open circuit potential (OCP) measurements, linear potentiodynamic polarization and cyclic voltammetry were performed. Linear potentiodynamic polarization was conducted in the potential range between the open circuit potential and −0.6 V (vs. SCE) for the cathodic scan and between the open circuit potential and 0.1 V (vs. SCE) for the anodic scan. The cyclic voltammetry curves were recorded in the potential range from −1 V (vs. SCE) to 1 V (vs. SCE). The scan rate used for linear potentiodynamic measurements was 1 mV/s, and 10 mV/s was used for cyclic voltammetric measurements.

NaCl (VWR BDH Prolabo), adenine (AD) (Sigma Aldrich), salicylaldoxime (SA) (Sigma Aldrich) and 4(5)-methylimidazole (4-(5)-MI) (Sigma Aldrich) were used for the preparation of suitable solutions in this investigation.

The effect of brass pretreatment in aqueous inhibitor solution (5·10^−3^ M AD, 5·10^−3^ M SA and 1·10^−2^ M 4(5)-MI) was also investigated.

### Surface analysis

The surfaces of brass coupons were tested after 24 h treatment in NaCl solution without and with the addition of 5∙10^−3^ M adenine. The surface morphology of brass coupons was examined by scanning electron microscopy (SEM, Tescan VEGA 3 LM) rigged with an Oxford EDS X-act Inca 350 system.

## Results and Discussion

### Open circuit potential measurements of brass

**Figure 1:**
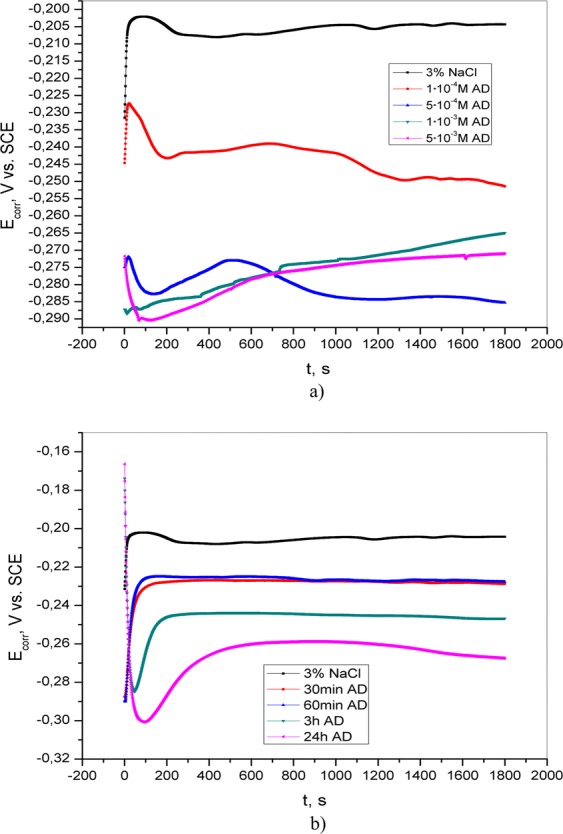
Open circuit potential of brass in 3% NaCl (**a**) without and with the addition of various concentrations of adenine; (**b**) after varying exposure times (30 min, 60 min, 3 h and 24 h) of brass in inhibitor solution.

**Figure 2:**
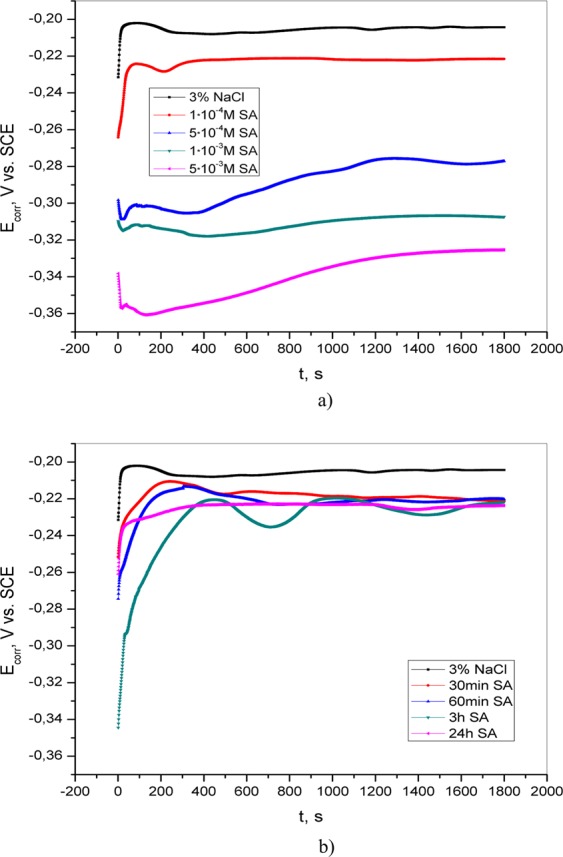
Open circuit potential of brass in 3% NaCl (**a**) without and with the addition of various concentrations of salicylaldoxime; (**b**) after varying exposure times (30 min, 60 min, 3 h and 24 h) of brass in inhibitor solution.

**Figure 3:**
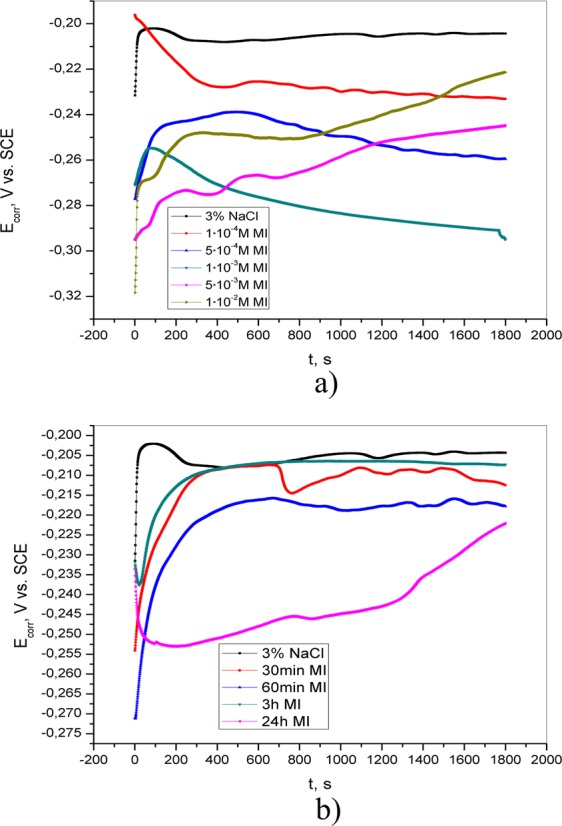
Open circuit potential of brass in 3% NaCl (**a**) without and with the addition of various concentrations of 4(5)-methylimidazole; (**b**) after varying exposure times (30 min, 60 min, 3 h and 24 h) of brass in inhibitor solution.

The open circuit potential of brass was measured in 3% NaCl solution without and with the addition of adenine, salicylaldoxime and 4(5)-methylimidazole. The obtained results are shown in Figs 1a–3a. In the inhibitor-free solution, the OCP shifts toward a negative direction at the beginning of immersion, indicating the deposition of corrosion products on the brass surface. The open circuit potential values are shifted in the negative direction as the concentration of inhibitor is increased. This behavior may be associated with the adsorption of inhibitor molecules on active sites on the surface of the brass^17,18^. The shift in OCP value due to increased concentration of the inhibitor is not significant, so it can be assumed that the tested compounds behave like mixed-type inhibitors, which will be discussed later. After the pretreatment of brass in the appropriate aqueous solution, the OCP was measured again (Figs 1b–3b). The obtained values are more negative than the value for blank solution. By comparing the OCP values of brass in 3% NaCl in the presence of 5·10^−3^ M AD, 5·10^−3^ M SA, and 1·10^−2^ M MI with those recorded after pretreatment, it can be seen that in the second case, the OCP values are more positive. This could be explained by the adsorption of inhibitors on the metal surface.

### Cyclic voltammetry measurements of brass

The initial contact of brass (Cu37Zn) with 3% NaCl solution leads to the formation of oxide layers via the following reactions^19^:

1 $${\rm{Zn}}+{{\rm{O}}}_{2}+4{{\rm{H}}}^{+}={{\rm{Zn}}}^{2+}+2{{\rm{H}}}_{2}{\rm{O}}+{{\rm{2e}}}^{-}$$

2 $$2{{\rm{Zn}}}^{2+}+2{{\rm{H}}}_{2}{\rm{O}}=2{\rm{ZnO}}+4{{\rm{H}}}^{+}$$

3 $${\rm{Cu}}\,\mbox{--}\,{{\rm{e}}}^{-}={{\rm{Cu}}}^{+}$$

4 $${{\rm{Cu}}}^{+}\,\mbox{--}\,{{\rm{e}}}^{-}={{\rm{Cu}}}^{2+}$$

5 $$2{{\rm{Cu}}}^{+}+{{\rm{H}}}_{2}{\rm{O}}={{\rm{Cu}}}_{2}{\rm{O}}+2{{\rm{H}}}^{+}$$

Furthermore, in the presence of chloride ions, the formation of CuCl species occurs according to reaction (6)^5^:

6 $${{\rm{Cu}}}^{+}+{{\rm{Cl}}}^{-}={\rm{CuCl}}$$

This layer has poor adhesion and has no ability to protect the metal. Furthermore, the layer is transformed into CuCl_2_^−^ via reaction (7):

7 $${\rm{CuCl}}+{{\rm{Cl}}}^{-}={{{\rm{CuCl}}}_{{\rm{2}}}}^{-}$$

The cathodic reaction of brass in the investigated solution is oxygen reduction according to reaction (8)^20^:

8 $${{\rm{O}}}_{2}+2{{\rm{H}}}_{2}{\rm{O}}+4{{\rm{e}}}^{-}=4{{\rm{OH}}}^{-}$$

The anodic current peak obtained in 3% NaCl solution without inhibitor suggests that copper undergoes oxidation and forms oxidation products.

**Figure 4:**
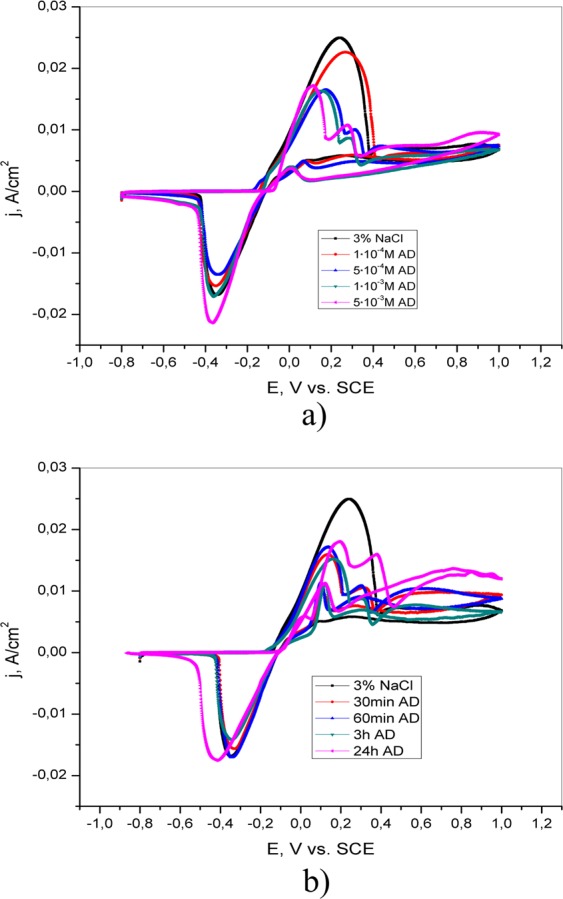
Cyclic voltammetric curves of brass in 3% NaCl (**a**) in the absence and presence of different concentrations of adenine; (**b**) after brass pretreatment in 5·10^−3^ M AD for different periods of time (30 min, 60 min, 3 h and 24 h).

Additionally, in accordance with the CV curves shown in Fig. 4a,b, two oxidation peaks can be observed in the presence of adenine (5·10^−4^ M, 1·10^−3^ M, and 5·10^−3^ M) in solution as well as in 3% NaCl after pretreatment in 5·10^−3^ M AD solution for different periods of time (30 min, 60 min and 3 h). The first peak corresponds to the oxidation of Cu to Cu^+ ^^21,22^. The intensity of this current peak is reduced as the concentration of adenine or the exposure time of brass to AD solution increases, indicating the protective ability of AD. In the cathodic direction, the reduction peak is observed at ~ −0.4 V vs. SCE, indicating the reduction of brass corrosion products. The second anodic current peak appears only if adenine is present either in the test solution or on the surface of the electrode. The peak intensity is greatest in the presence of the highest adenine concentration or after the longest pretreatment. This current peak can be attributed to the reaction of Cu^+^ and adenine and the formation of the Cu(I)-adenine complex^23^.

It is known that the form of adenine in neutral NaCl solution is anionic^13^. This form of adenine forms a Cu(I)-adenine complex with Cu^+ ^^24^. At lower concentrations adenine reacts with CuCl and forms the following compound on the electrode surface^13^:

9 $${{\rm{CuCl}}}_{{\rm{ads}}}+{{\rm{C}}}_{5}{{\rm{H}}}_{5}{{\rm{N}}}_{5}={[{\rm{Cu}}-{{\rm{C}}}_{5}{{\rm{H}}}_{4}{{\rm{N}}}_{5}]}_{{\rm{ads}}}+{{\rm{Cl}}}^{-}+{{\rm{H}}}^{+}$$

However, with the addition of a higher concentration of adenine, adenine adsorbs directly on the electrode surface according to the following reaction^13^:

10 $${\rm{Cu}}+{{\rm{C}}}_{5}{{\rm{H}}}_{4}{{\rm{N}}}_{5}-{{\rm{e}}}^{-}={[{\rm{Cu}}-{{\rm{C}}}_{5}{{\rm{H}}}_{4}{{\rm{N}}}_{5}]}_{{\rm{ads}}}$$

As a result of these reactions, a protective layer [Cu-C_5_H_4_N_5_]_ads_ is formed on the electrode surface. According to Sharma and Trnkova^23^, it is also possible to form adenine dimers at higher potentials in neutral and alkaline media.

**Figure 5:**
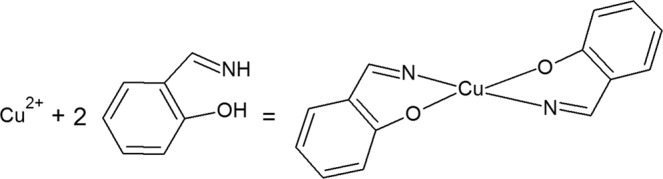
Formation of complex by copper ions and salicylaldoxime.

**Figure 6:**
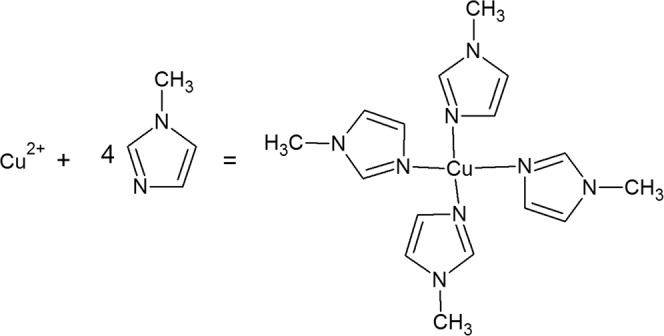
Formation of complex by copper ions and 4(5)-methylimidazole.

**Figure 7:**
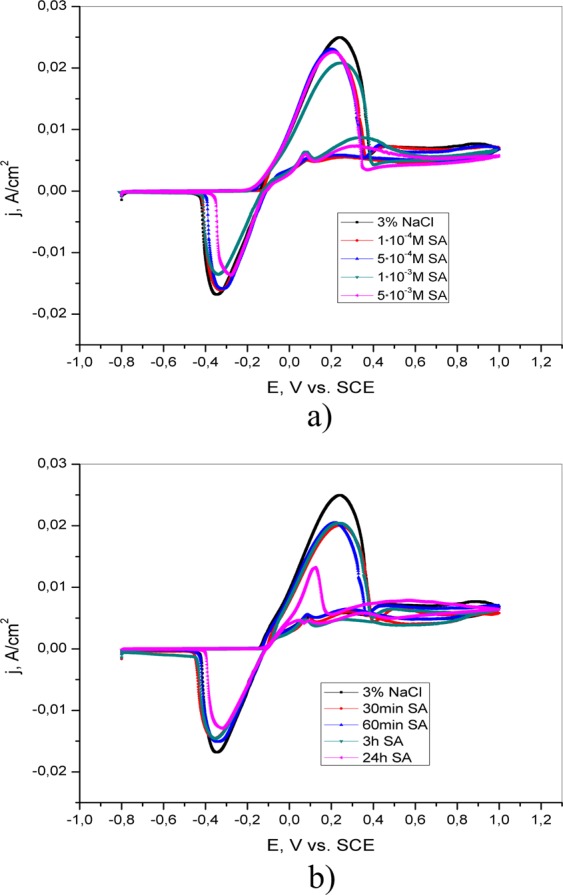
Cyclic voltammetric curves of brass in 3% NaCl (**a**) in the absence and presence of different concentrations of salicylaldoxime; (**b**) after brass pretreatment in 5·10^−3^ M SA for different periods of time (30 min, 60 min, 3 h and 24 h).

According to literature data Cu^2+^ react with salicylaldoxime and 4(5)-methylimidazole and form complexes^25–30^. Formation of complexes is present in Figs 5 and 6. In accordance with the literature, salicylaldoxime has the ability to form insoluble complexes with copper^31^. The formation of this layer on the brass surface prevents further corrosion and prevents the adsorption of aggressive ions on the brass surface. The CV curves in the presence of salicylaldoxime are similar to that of brass in inhibitor-free solution (Fig. 7a), and the mechanism of brass dissolution is not changed. The addition of SA reduces the intensity of the cathodic peak to a certain extent, suggesting an inhibitory effect. The cyclic voltammetry curves of brass after pretreatment in 5·10^−3^ M SA (Fig. 7b) indicate a decrease in both the anodic and cathodic current peaks with increasing immersion time. A significant reduction in these peaks is evident after the longest pretreatment, indicating a strong interaction between SA and the brass surface. This behavior could be explained by the formation of a compact protective layer on the brass surface.

**Figure 8:**
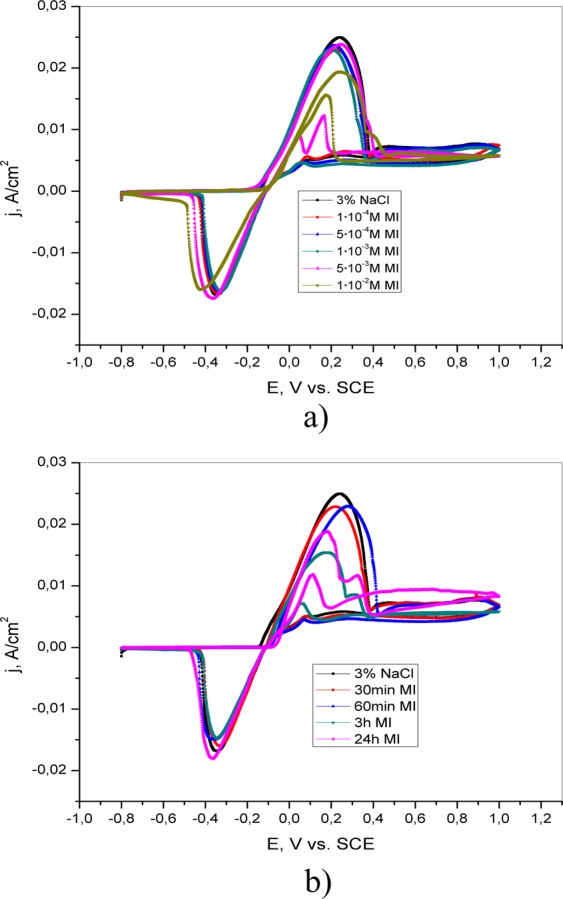
Cyclic voltammetric curves of brass in 3% NaCl (**a**) in the absence and presence of different concentrations of 4(5)-methylimidazole; (**b**) after brass pretreatment in 1·10^−2^ M 4(5)-MI for different periods of time (30 min, 60 min, 3 h and 24 h).

The addition of 1·10^−2^ M 4(5)-methylimidazole causes a reduction in the anodic current peak, indicating the formation of a protective layer on the brass surface, which effectively prevents the dissolution of the brass (Fig. 8a). It is assumed that the presence of the inhibitor at concentrations less than 1·10^−2^ M was not sufficient to form a protective layer and that aggressive ions came into contact with the brass. The mechanism of formation of the protective film can be described by reaction (11)^32^:

11 $${\rm{Cu}}+{\rm{inh}}={\rm{Cu}}{({\rm{inh}})}_{{\rm{ads}}}={{\rm{Cu}}}^{{\rm{n}}+}+{{\rm{ne}}}^{-}+{\rm{inh}}$$

The formed Cu(inh)_ads_ does not fully cover the surface of the brass at lower concentrations of the inhibitor. Therefore, the dissolution of metals occurs in places on the brass surface that are not covered with the Cu(inh)_ads_ layer.

During the reverse scan, a small activation anodic peak is observed in the presence of SA and 4(5)-MI, indicating the occurrence of the reactivation process. It is proposed that the brass surface in 3% NaCl solution is not completely covered by a protective layer of inhibitor. The current density of the activation peak is the highest in the presence of 4(5)-methylimidazole. This may be explained by either the formation of porous and thin protective films^33^ or competition between dissolution and precipitation of the layer on the brass surface^34^.

The influence of pretreatment of the electrode in 4(5)-MI aqueous solution is shown in Fig. 8b. The current densities of both the anodic and cathodic peaks are affected by immersion time. Increasing the exposure time of brass in 4(5)-MI solution (3 h and 24 h) leads to the appearance of two anodic peaks. Similar behavior is obtained after the exposure of brass to AD solution. This behavior could be explained by the reaction of Cu^+^ with 4(5)-MI and the formation of a Cu(I)-imidazole complex. The formed Cu(I)-imidazole complex has anticorrosive properties and prevents the further reaction of chloride ions and brass, which is in agreement with the literature^35,36^.

### Potentiodynamic polarization measurements of brass

**Figure 9:**
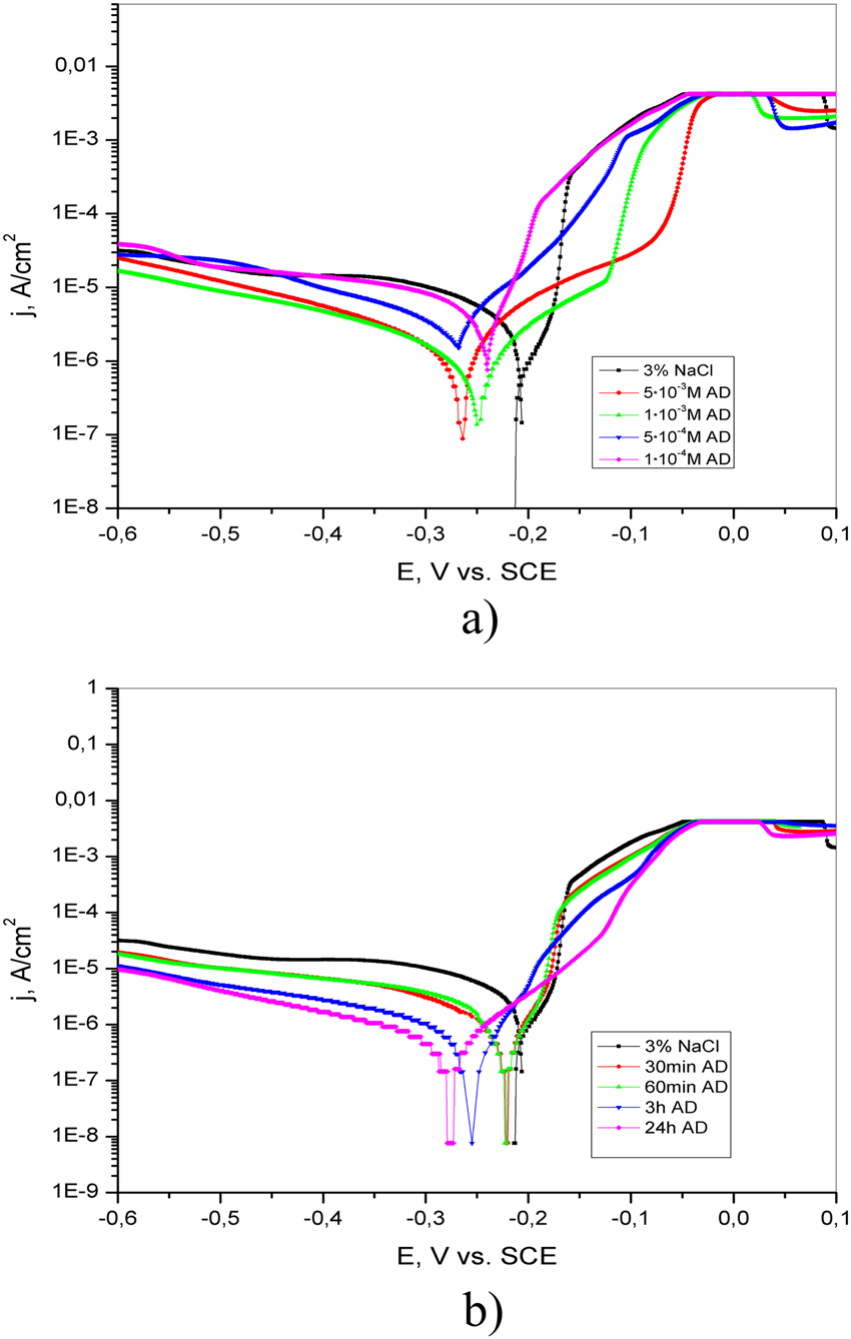
Potentiodynamic polarization curves of brass in 3% NaCl (**a**) in the absence and presence of different concentrations of adenine; (**b**) after brass pretreatment in 5·10^−3^ M AD for different periods of time (30 min, 60 min, 3 h and 24 h).

**Figure 10:**
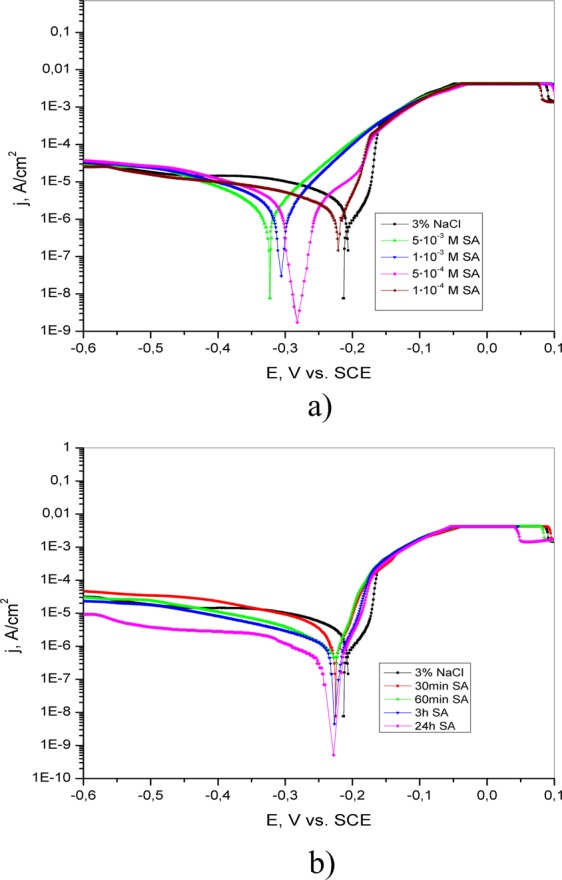
Potentiodynamic polarization curves of brass in 3% NaCl (**a**) in the absence and presence of different concentrations of salicylaldoxime; (**b**) after brass pretreatment in 5·10^−3^ M SA for different periods of time (30 min, 60 min, 3 h and 24 h).

**Figure 11:**
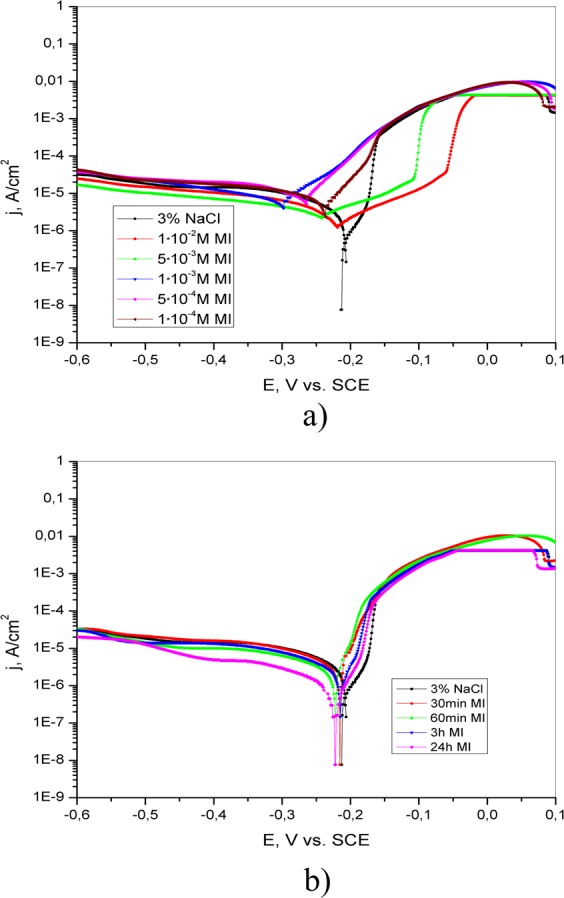
Potentiodynamic polarization curves of brass in 3% NaCl (**a**) in the absence and presence of different concentrations of 4(5)-methylimidazole; (**b**) after brass pretreatment in 1·10^−2^ M 4(5)-MI for different periods of time (30 min, 60 min, 3 h and 24 h).

The polarization curves of brass in 3% NaCl without and with the addition of different concentrations of 4(5)-methylimidazole, salicylaldoxime and adenine are shown in Figs 9a–11a, respectively. The presence of the investigated inhibitors leads to a decrease in the corrosion current density relative to that of inhibitor-free solution. This can be explained by the formation of a protective layer on the brass surface.

The addition of adenine causes a change in brass behavior in 3% NaCl solution. This change is illustrated in Fig. 9a. In the presence of 1·10^−4^ M adenine, the anodic current density rapidly increases with potential. It is proposed that this concentration of adenine is not enough to form a protective layer (to cover the entire brass surface). Upon further increasing the adenine concentration, both the anodic and cathodic current densities are reduced in comparison to those in blank solution, and the corrosion potential (E_corr_) values shift toward the negative direction.

The corresponding electrochemical parameters of brass corrosion in 3% NaCl, including the corrosion potential (E_corr_), corrosion current density (j_corr_), anodic (b_a_) and cathodic (b_c_) Tafel slopes and inhibition efficiency (IE), were calculated according to polarization curves. The inhibition efficiency was determined via Eq. (12):

12 $$ \% IE=(({j}_{corr}-{j}_{corr(inh)})/{j}_{corr})\cdot 100$$

where *j*_*corr*_ and *j*_*corr(inh)*_ are corrosion current densities without and with the addition of inhibitor in 3% NaCl solution.

As shown in Table 1, the corrosion current densities are decreased in the presence of inhibitors, indicating an inhibitory effect. Additionally, the anodic (b_a_) and cathodic (b_c_) Tafel slopes are changed in the presence of adenine (Table 1). This behavior can be explained by the formation of a protective layer on the metal surface^11^.

**Table 1:** Electrochemical parameters of brass corrosion in 3% NaCl solution without and with the addition of different concentrations of adenine and after pretreatment in adenine solutions for various time periods.

Inhibitor, M	Immersion time, min	E_corr_, V vs. SCE	j_corr_, A/cm^2^	−b_c_, V	b_a_, V	IE, %
3% NaCl	/	−0.215	8.03·10^−6^	0.518	0.078	/
1·10^−4^	/	−0.241	2.70·10^−6^	0.150	0.040	66.4
5·10^−4^	/	−0.269	2.52·10^−6^	0.197	0.089	68.4
1·10^−3^	/	−0.250	7.22·10^−7^	0.133	0.078	91.0
5·10^−3^	/	−0.266	6.27·10^−7^	0.066	0.057	92.2
	30	−0.221	5.17·10^−7^	0.092	0.042	93.6
	60	−0.221	3.91·10^−7^	0.045	0.038	95.1
	180	−0.255	1.78·10^−7^	0.047	0.038	97.7
	1440	−0.278	1.40·10^−7^	0.061	0.048	98.3

/(In the immersion time column) - means that the measurements were performed without pretreatment of brass electrode in inhibitor solution.

/(In the IE column) - means that there is no inhibition activity.

Potentiodynamic polarization curves of brass obtained in 3% NaCl solution after immersion in 5·10^−3^ M AD, 5·10^−3^ M SA and 1·10^−2^ M 4(5)-MI for different periods of time are presented in Figs 9b–11b, respectively.

According to Fig. 9b, the cathodic current density decreases after pretreatment of brass for 30 min in 5·10^−3^ M AD relative to that of inhibitor-free solution. Furthermore, as the exposure time increases, the anodic current density also decreases, indicating a decrease in the brass dissolution processes. It is evident that IE increases as the immersion time of the brass electrode in AD solution increases (Table 1). This can be explained by the formation of a more stable protective layer on the brass surface under the examined conditions, thereby protecting the brass from corrosion. It is evident from Table 1 that the values of E_corr_ are shifted in the negative direction as immersion time increases. This displacement of corrosion potential is lower than 85 mV, indicating that adenine behaves like a mixed-type inhibitor.

In the presence of SA, the corrosion potential (E_corr_) is moved toward less positive values in comparison to that of blank solution, but there is no definite shift in E_corr_ (Fig. 10). The addition of SA leads to a significant decrease in the cathodic current density compared with the anodic current density, especially in the vicinity of the corrosion potential. It is proposed that SA acts as a mixed-type inhibitor with a pronounced effect on the cathodic reaction. The calculated parameters in Table 2 indicate that the corrosion current density decreases with increasing SA concentration. Additionally, the values of the anodic and cathodic Tafel slopes are changed in the presence of SA, indicating the formation of a protective layer on the metal surface.

**Table 2:** Electrochemical parameters of brass corrosion in 3% NaCl solution without and with the addition of different concentrations of salicylaldoxime and after pretreatment in salicylaldoxime solutions for various time periods.

Inhibitor, M	Immersion time, min	E_corr_, V vs. SCE	j_corr_, A/cm^2^	−b_c_, V	b_a_, V	IE, %
3% NaCl	/	−0.215	8.03·10^−6^	0.518	0.078	/
1·10^−4^	/	−0.221	3.78·10^−6^	0.241	0.040	52.9
5·10^−4^	/	−0.284	3.02·10^−6^	0.077	0.082	62.4
1·10^−3^	/	−0.306	2.11·10^−6^	0.057	0.045	73.7
5·10^−3^	/	−0.325	1.34·10^−6^	0.051	0.047	83.3
	30	−0.225	9.13·10^−7^	0.045	0.025	88.6
	60	−0.225	7.25·10^−7^	0.086	0.022	91.0
	180	−0.226	5.00·10^−7^	0.059	0.028	93.8
	1440	−0.228	2.02·10^−7^	0.062	0.023	97.5

/(In the immersion time column) - means that the measurements were performed without pretreatment of brass electrode in inhibitor solution.

/(In the IE column) - means that there is no inhibition activity.

The electrochemical parameters of brass corrosion in 3% NaCl after pretreatment in aqueous SA solution for various periods of time are also presented in Table 2. The shift in E_corr_ is not significant with increasing immersion time, but the value is more positive than that obtained in 5·10^−3^ M SA without pretreatment. This behavior indicates that SA acts as a mixed-type inhibitor. Additionally, the pretreatment of brass in 5·10^−3^ M SA solution leads to a remarkable decrease in corrosion current density relative to the value obtained in 5·10^−3^ M SA without pretreatment.

Potentiodynamic polarization curves of brass in 3% NaCl solution without and with the addition of 4(5)-MI are shown in Fig. 11. Similar to adenine, the presence of 4(5)-MI leads to the displacement of both the anodic and cathodic curves toward lower current values. In accordance with this behavior, it can be said that 4(5)-MI acts as a mixed-type inhibitor. The electrochemical parameters presented in Table 3 indicate that the inhibition efficiency increases as the concentration of 4(5)-MI increases. The values of the anodic and cathodic Tafel slopes are changed in the presence of inhibitors, suggesting that a protective layer is formed on the brass surface^11^.

**Table 3:** Electrochemical parameters of brass corrosion in 3% NaCl solution without and with the addition of different concentrations of 4(5)-methylimidazole and after pretreatment in adenine solutions for various time periods.

Inhibitor, M	Immersion time, min	E_corr_, V vs. SCE	j_corr_, A/cm^2^	−b_c_, V	b_a_, V	IE, %
3% NaCl	/	−0.215	8.03·10^−6^	0.518	0.078	/
1·10^−4^	/	−0.239	6.44·10^−6^	0.175	0.064	19.8
1·10^−3^	/	−0.299	6.04·10^−6^	0.235	0.084	24.8
5·10^−3^	/	−0.243	3.05·10^−6^	0.345	0.164	62.0
1·10^−2^	/	−0.220	2.10·10^−6^	0.116	0.163	73.8
	30	−0.216	1.72·10^−6^	0.075	0.022	78.6
	60	−0.222	9.20·10^−7^	0.043	0.013	88.5
	180	−0.216	8.88·10^−7^	0.038	0.027	88.9
	1440	−0.222	4.57·10^−7^	0.070	0.033	94.3

/(In the immersion time column) - means that the measurements were performed without pretreatment of brass electrode in inhibitor solution.

/(In the IE column) - means that there is no inhibition activity.

As presented in Table 3, the values of E_corr_ do not change significantly after the immersion of brass in aqueous 4(5)-MI solution. Additionally, Fig. 11b shows that the pretreatment of the electrode causes a remarkable decrease in the corrosion rate, shifting the cathodic curves to lower current densities. This can be explained by the formation of a protective layer on the brass surface that hinders the cathodic reaction. It is proposed that the stability of the formed layer increases with exposure time, leading to increased IE.

The results obtained by potentiodynamic polarization measurements confirm that the protective ability of the inhibitors increases with increasing inhibitor concentration as well as with increasing immersion time. Analysis of the curves presented in Figs 9–11 leads to the conclusion that better anticorrosive protection is provided by electrode pretreatment in inhibitor solution than by the addition of inhibitor to the test solution. However, with the potential increase curves obtained in the NaCl solution with and without addition of inhibitor are coincide, which indicates the dissolution of the protective film and the desorption of inhibitor molecules from the surface of the electrode. By comparing the anodic and cathodic Tafel slopes obtained without and with the appropriate pretreatment of brass (Tables 1–3), it can be said that both b_a_ and b_c_ are generally lower in the second case. This probably indicates that the pretreatment of brass in the appropriate solution leads to the formation of a compact protective layer. Additionally, the obtained results indicate that the tested inhibitors remarkably reduce the corrosion rate of brass in 3% NaCl solution by hindering both reactions^37^.

### Surface analysis

**Figure 12:**
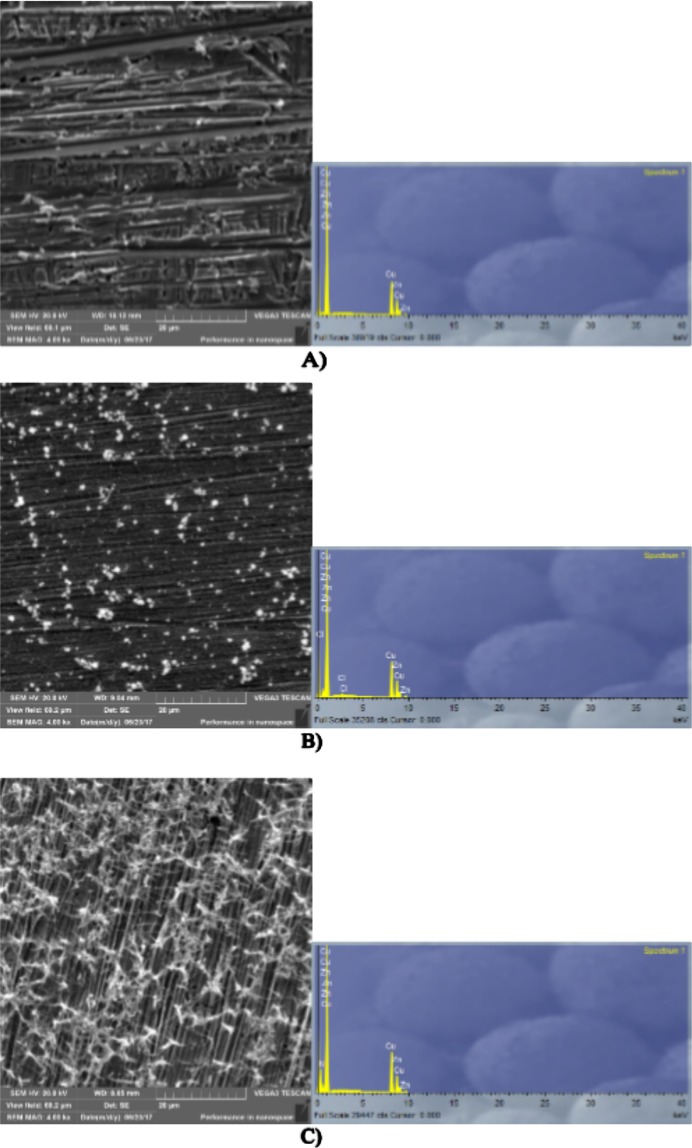
SEM images and EDS spectra of brass surfaces (**A**) without any treatment; (**B**) after 24 h immersion in 3% NaCl solution; (**C**) after 24 h immersion in 3% NaCl solution with the addition of 5·10^−3^ M AD.

**Figure 13:**
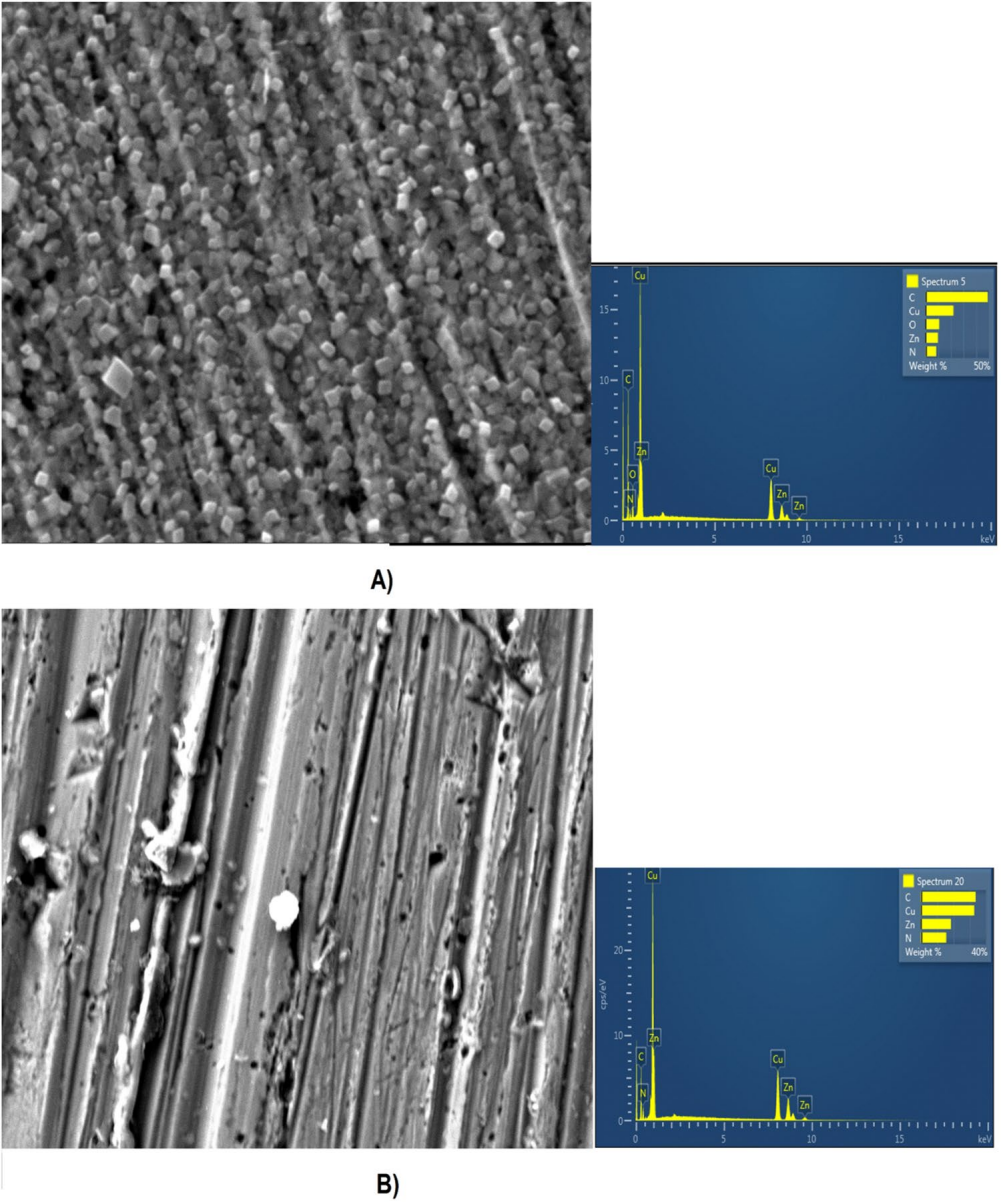
SEM images and EDS spectra of brass surfaces (**A**) after 24 h immersion in 3% NaCl solution with the addition of 5·10^−3^ M SA; (**B**) after 24 h immersion in 3% NaCl solution with the addition of 1·10^−2^ M 4(5)-MI.

Figures 12 and 13 show SEM-EDS images of polished brass surfaces without any treatment and after the immersion of the electrode for 24 h in 3% NaCl, 5·10^−3^ M AD, 5·10^−3^ M SA and 1·10^−2^ M 4(5)-MI solutions.

According to the figure, the brass surface after pretreatment in NaCl solution is different from the brass surface without any treatment. EDS analysis shows that Cl^−^ ions exist on the brass surface, which indicates that corrosion products are formed. Additionally, after pretreatment in adenine, salicylaldoxime and 4(5)-methylimidazole solutions, the electrode surface is changed. Moreover, on the brass surface presented in Fig. 12B, there exist adsorbed species that are not found on the clean surface (Fig. 12A). The EDS spectrograms show that nitrogen atoms are detected on the brass surface after treatment of the electrode in inhibitor solutions. Furthermore, nitrogen atoms derive from adenine, salicylaldoxime and 4(5)-methylimidazole molecules, and their presence indicates that the adsorption of AD, SA and 4(5)-MI occur at the active sites on the brass surface (Figs 12C and 13A,B).

### Adsorption isotherm

The important step in the inhibition of corrosion is the adsorption of inhibitors on the metal surface. Additionally, the adsorption isotherm provides information about the interaction between the metal surface and a given inhibitor. To obtain information about the mode of corrosion inhibition of brass by adenine, salicylaldoxime and 4(5)-methylimidazole, the Langmuir adsorption model was examined. This adsorption model is given by Eq. (13):

13 $$C/\theta =1/K+C$$

**Figure 14:**
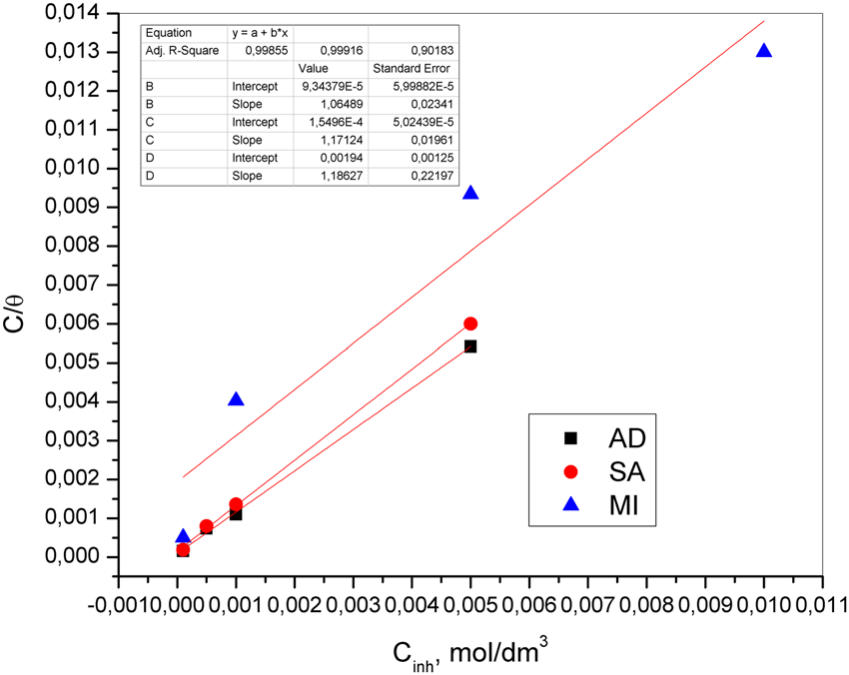
Langmuir adsorption isotherms for brass in 3% NaCl containing different concentrations of adenine, salicylaldoxime and 4(5)-methylimidazole.

The obtained linear relation of c/θ versus inhibitor concentration (Fig. 14) suggested that the Langmuir adsorption isotherm provides the best description of the adsorption behavior of the investigated compounds on the brass surface.

The Gibbs free energy (−ΔG) can be calculated according to Eq. (14):

14 $$-\Delta G=(lnK-\,\mathrm{ln}(1/55.55))\cdot R\cdot T$$

where R is the universal gas constant, T is the thermodynamic temperature, and the value of 55.5 is the molar concentration of water in solution.

The linear regression coefficient (R^2^) is almost equal to 1, indicating that the adsorbed molecules occupy only one site. The obtained Gibbs free energies present in Table 4 point to stronger adsorption of adenine and salicylaldoxime than 4(5)-methylimidazole.

**Table 4:** Adsorption parameters of adenine, salicylaldoxime and 4(5)-methylimidazole on the brass surface in 3% NaCl.

Inhibitor	1/K	slope	R^2^	-ΔG, kJ/mol
AD	0.00009563	1.06	0.998	32.3
SA	0.00015514	1.17	0.999	31.1
4(5)-MI	0.00163	1.21	0.949	25.4

### Quantum chemical calculation

Quantum chemical calculations and molecule geometry optimization were performed using ArgusLab 4.0^38^, software previously proven useful for this purpose^39–41^. The PM3-SCF method was applied. The calculated parameters are summarized in Table X and include the highest occupied molecular orbital energy (E_HOMO_), lowest unoccupied molecular orbital energy (E_LUMO_), energy gap (ΔE = E_LUMO_ − E_HOMO_) and dipole moment of inhibitor (μ). Further calculations using the obtained parameters included the determination of the ionization potential (I), electron affinity (A), electronegativity (χ), global hardness (η) and the maximum number of electrons transferred (ΔN_max_). The following Eqs (15–19) were used:

15 $$I=-\,{E}_{HOMO}$$

16 $$A=-\,{E}_{LUMO}$$

17 $${\rm{\chi }}=0.5\cdot (I+A)$$

18 $${\rm{\eta }}=0.5\cdot (I-A)$$

19 $$\Delta {N}_{max=}{\rm{\chi }}/(2{\rm{\eta }})$$

**Figure 15:**
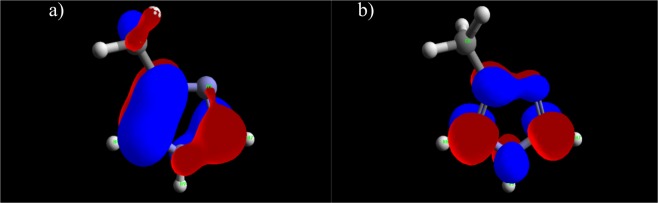
Distribution of HOMO (**a**) and LUMO (**b**) of 4(5)-methylimidazole.

**Figure 16:**
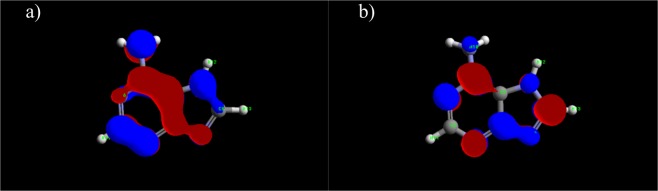
Distribution of HOMO (**a**) and LUMO (**b**) of adenine.

**Figure 17:**
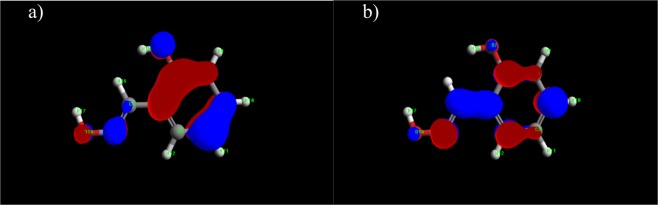
Distribution of HOMO (**a**) and LUMO (**b**) of salicylaldoxime.

The proposed spatial distribution of HOMO and LUMO is presented in Figs 15–17. The values of the quantum chemical parameters calculated for methylimidazole, adenine and salicylaldoxime are in accordance with the available previously published results^42–44^.

Comparative analysis of the experimental results and calculation results indicates that several quantum chemical parameters can be correlated with inhibition efficiency. Namely, as the energy gap decreases, organic molecules can provide higher inhibition efficiency. Higher values of E_HOMO_ and lower values of E_LUMO_ are, in this case, also good indicators of potential inhibition efficiency. These values are indicators of the tendency of inhibitor molecules to donate (E_HOMO_) or accept (E_LUMO_) electrons and hence to be adsorbed on the metal surface (ΔE). A similar trend can be observed regarding molecular hardness; i.e., as the molecular hardness decreases, the molecule reacts with the surface more readily, and the corrosion effect decreases. Similar observations have been presented in the literature^45–48^. The maximum number of electrons transferred increases in the same order as the experimentally obtained inhibition efficiency, as was also noticed by Bedair^49^. The molecule with the highest dipole moment also provides the highest inhibition efficiency, which is in accordance with results found in the literature as well^48,50^. Nevertheless, all the studied compounds have dipole moment values greater than that of water (1,88 D), indicating strong interactions between the inhibitors and the metal surface^51^. Under the investigated conditions, adenine provided the best inhibition effect among the studied compounds, which can also be expected based on the quantum chemical parameters presented in Table 5.

**Table 5:** Quantum chemical parameters.

	Methylimidazole	Adenine	Salicylaldoxime
E_HOMO,_ eV	−9.221	−9.162	−9.218
E_LUMO,_ eV	0.730	−0.705	−0.534
ΔE, eV	9.951	8.457	8.684
μ, D	3.63678763	5.89602736	1.95167390
I, eV	9.221493712	9.162334824	9.21847318
A, eV	−0.73009796	0.705416676	−0.533600108
χ, eV	4.245697876	4.93387575	4.342436536
η, eV	4.975795836	4.228459074	4.876036644
ΔN_max_	0.426635056	0.583412971	0.445283419

## Conclusion

The influence of different concentrations of adenine, salicylaldoxime and 4(5)-methylimidazole on the corrosion behavior of brass in 3% NaCl was studied.

Cyclic voltammetric and potentiodynamic polarization measurements showed that the investigated compounds can be successfully used as brass corrosion inhibitors in 3% NaCl solution. The inhibition efficiency of the investigated compounds depends on their concentrations in solution. Additionally, the exposure time of brass in the appropriate solution is an important step in protecting brass against corrosion. The adsorption of the inhibitors on the brass surface obeys the Langmuir adsorption isotherm. The quantum chemical calculations are in good agreement with the results obtained by electrochemical measurements.
